# Do inattention and hyperactivity symptoms equal scholastic impairment? evidence from three European cohorts

**DOI:** 10.1186/1471-2458-7-327

**Published:** 2007-11-13

**Authors:** Alina Rodriguez, Marjo-Riitta Järvelin, Carsten Obel, Anja Taanila, Jouko Miettunen, Irma Moilanen, Tine Brink Henriksen, Katri Pietiläinen, Hanna Ebeling, Arto J Kotimaa, Karen Markussen Linnet, Jørn Olsen

**Affiliations:** 1 Department of Psychology, Uppsala University, Sweden; 2 Department of Public Health Science and General Practice, University of Oulu, Finland; 3 Department of Epidemiology and Public Health, Imperial College London, London, UK; 4 Deptartment of Pediatrics, Aarhus University Hospital, Skejby, Denmark; 5 Danish Epidemiology Science Centre, Aarhus University, Denmark; 6 Department of Epidemiology, School of Public Health, UCLA, Los Angeles, USA; 7 Clinic of Child Psychiatry, University and University Hospital of Oulu, Finland; 8 Department of Psychiatry, University and University Hospital of Oulu, Finland

## Abstract

**Background:**

Attention Deficit/Hyperactivity Disorder (ADHD) affects many children, adolescents, and adults and is associated with a number of impairments. Poor academic performance is related to ADHD in clinical samples. However, it is unclear to what extent core ADHD symptoms and scholastic impairment are related in non-referred school-aged children.

**Methods:**

Data come from three population-based cohorts from Sweden, Denmark, and Finland, which are part of the Nordic Network on ADHD. The combined sample size was 13,087 children who were studied at ages 7–8 or 10–12 years. Teachers rated children on inattention and hyperactivity symptoms and reported children's scholastic performance on basic skills.

**Results:**

There was a significant association in all cohorts between core ADHD symptoms and scholastic impairment in reading, writing, and mathematics. Particularly, inattention was related to a two to tenfold increase in scholastic impairment. Prevalence of hyperactivity symptoms was similar across the three cohorts, but inattention was lowest among children from the Finnish cohort, after stratification on living conditions.

**Conclusion:**

These results extend previous reports of scholastic impairment among children with clinically diagnosed ADHD to non-referred population samples from three European countries. Surveillance policies should be implemented in school systems to catch children in need of behavioral or scholastic support early.

## Background

Attention Deficit/Hyperactivity Disorder (ADHD) is the most common neurobehavioral disorder in children and adolescents. ADHD is associated with impairment in all aspects of a child's life, i.e. family, social, and academic [1,2]. Children with ADHD often follow a sustained negative developmental trajectory [3] and impairments can continue into adulthood [4]. ADHD is a concern for public health and policy makers not only due to the wide ranging associated difficulties, but also because it is a substantial economic burden for society in terms of medical treatment [5] and indirect costs related to high risk behaviors [6,7] and their consequences. Early identification of possible cases would be helpful in the planning of public services.

ADHD is characterized by inattention and hyperactivity symptoms inappropriate for age or developmental level and diagnosis requires symptoms to be associated with considerable impairment. Scholastic underachievement is associated with clinically diagnosed ADHD in children [8] and lower academic attainment and lower socioeconomic status in adults [4]. However, it is unknown whether the relation between scholastic impairment also holds true for core ADHD symptoms in the much larger group of cases that are not clinically diagnosed. If ADHD symptoms are trait-like and continuously distributed in the population as has been previously proposed [9], then we expect that a positive association will be evident between core symptoms and impairment, as is true for diagnosed cases. If the presence of core symptoms, irrespective of diagnosis, is systematically associated with scholastic impairment, then teachers may be key in early identification of children who need support.

Our aim was to investigate the association between core symptoms of ADHD and impairments in basic scholastic skills, i.e. reading, writing, and mathematics in large non-referred samples from three European countries. This approach advances our understanding in several ways. First, we examine the basic academic skills, as opposed to concentrating on reading, which has been previously associated with ADHD. Second, the large sample size enables us to adequately test whether the relations are consistent for girls as well as boys. Third, we have the opportunity to check whether findings replicate in the three participating countries.

We focused on teacher-rated restlessness, fidgetiness, and inattention because these symptoms are basic to the diagnostic criteria listed for ADHD in the DSM-IV [1] and Hyperkinetic Disorder in the International Classification of Diseases (ICD)-10 [10]. Further these symptoms appear in widely used parent, teacher, and self-administered screening instruments (e.g. Rutter Scale; Strengths and Difficulties Questionnaire, SDQ; Connors; Child Behavior Checklist, CBCL). ADHD behaviors at school, as opposed to at home, are especially important because previous work examining the full range of symptoms has found that they predict later academic underachievement [11]. Teacher ratings have been found to make a stronger contribution to the prediction of ADHD subtype than parent ratings [12] and have high classification accuracy of ADHD diagnosis [13].

We studied a large sample of children from three population based birth cohorts from Sweden, Denmark, and Finland. These countries share similar cultural traditions, political structures, and tax-paid healthcare, school systems and social services for children. Despite general similarities, local variations exist and this provides an opportunity to examine whether the associations replicate across a large geographic region. Most previous studies of ADHD have been confined to specific geographic locations. We hypothesized that the core ADHD symptoms and scholastic impairment would be positively related in all cohorts. Further, we expected to find cross-national similarity in the prevalence rates of these symptoms.

## Methods

### Samples

Prospective data originate from three birth cohorts from Sweden, 1^st ^Child in the Family, Denmark, The Aarhus Birth Cohort (ABC), and Finland, Northern Finland 1985–86 Birth Cohort (NFBC 1986), which longitudinally investigate medical and psychosocial endpoints. These cohorts are part of the Nordic Network on ADHD, which the Nordic Ministry of Health established and funded to promote a collaborative scientific effort to study various aspects of ADHD. The cohorts share a number of characteristics that make comparison reasonable, although the studies were not originally designed for cross-national comparison. The Ethics Review Board in each of the respective countries approved the studies.

All cohorts consecutively recruited women in early pregnancy via antenatal health services and achieved high recruitment rates (91% to 99%). Antenatal health care in the three countries is tax-paid. The percentage of pregnant women not receiving this type of antenatal care is less than 0.2% [14]. Routines for antenatal care are standardized within each country and all countries have low perinatal and infant mortality rates [15,16]. Inclusion criteria were the ability to understand the local language and additionally, in Sweden, nulliparity and Scandinavian origin. Participants received antenatal health care in either Uppsala County (Sweden), Department of Obstetrics and Gynecology in Aarhus (Denmark), or in Oulu or Lapland provinces (Finland). Antenatal care was provided at all levels as appropriate.

At follow-up participants were traced through the national registries using personal identification numbers in each country, enabling us to trace even those who moved outside the original geographic areas, i.e. anywhere within the national borders. Follow-up data collection occurred when children were approximately 7–8 years in Sweden and Finland and 10–12 years old in Denmark. Permission to contact the child's teacher was obtained from parents in all cohorts. All cohorts collected data on child behavior symptoms and scholastic performance from teachers via postal questionnaire.

### Variables

Teachers assessed child behavior using the official translations of the Strengths and Difficulties Questionnaire (SDQ) [17] in Sweden and Denmark and the Rutter scale (RB2) [18] in Finland. Scales were completed in full and in accordance with procedures for each instrument in the local language. The SDQ builds on the Rutter scale [19] and are highly correlated [17]. Both have documented reliability and validity [18,20] and cutoff scores on both instruments discriminate well between children with and without a clinical disorder [18,20]. SDQ assesses hyperactivity-inattention with a five-item subscale while the Rutter scale uses three items. The three items on the Rutter are essentially equivalent to three of the SDQ hyperactivity-inattention items and are the focus of this paper: SDQ items (nr. 2) restless, (nr. 10) fidgety, and (nr. 15) easily distracted and Rutter B2 items (nr. 1) restless (nr. 3) squirmy, fidgety, and (nr. 16) not able to concentrate. Behavioral descriptions are scored similarly on both instruments: *0 *(does not apply), *1 *(somewhat true), or *2 *(certainly true).

Teachers assessed deficits in reading, writing, and mathematical skills using 7-point and 5-point scales in Sweden and Denmark, respectively, and impaired/unimpaired format in Finland. We dichotomized the scales into below vs. average and above average performance for the purpose of the present study. Previous work shows that teachers using single-item ratings are accurate judges of impairment and their ratings concur with achievements test results [21,22]. Table 1 summarizes key participant characteristics and measures for the cohorts.

**Table 1:** Participants and measures

	Sweden	Denmark	Finland
**Initial data collection (years)**	1992 – 1994	1990–1992	1985 – 1986
Initiated during gestational week	10	14	12
Geographic area	Uppsala	Aarhus	Northern Finland
	County		(Oulu Lapland Provinces)
Inclusion criteria	nulliparity + Scandinavian origin	all pregnant women	all pregnant women
Sample size			
Mothers (% of eligible)	476 (91%)	8010 (98%)	9362 (99%)
live births	411	8244	9432
Maternal age (Y, sd)	27.0 (4)	28.6(5)	27.8 (6)
Family structure at birth			
cohabitated with expectant father	91%	95%	95%
**Follow-up data collection**	2001–2002	2001	1993–1994
Retained at follow-up	290 (74%)	5039 (61%)	9297 (99%)
Sample size:			
Participating eligible teachers^1^	208 (96%)	4354 (85%)	8525 (92%)
Child age (years)	7–8	10–12	7–8
Child gender (% boys)	49%	51%	51%
Maternal education (%)			
secondary	85.7	90.6	90.5
college/university^2^	15.2	9.5	9.5
Family structure			
two biological parents	78.1%	78.2%	87.8%
disrupted family^3^	21.9%	21.8%	12.2%
Inattention & hyperactivity symptoms assessment	SDQ^4^	SDQ^4^	CBQ^5^
Scholastic performance:			
writing, reading, mathematics	7-pt scale	5-pt scale	impaired/unimpaired

^1 ^teachers' eligiblity was determined by parental consent

^2 ^four or more years of college/university education or university degree

^3 ^disrupted family encompassed single parent households and reconstructed family with step-parent

^4 ^Strengths and Difficulties Questionnaire subscale for hyperactivity-inattention items: (nr. 2) restless, (nr. 10) fidgety, and (nr. 15) easily distracted

^5 ^Children's Behaviour Questionnaire Rutter B2 subscale for hyperactivity-inattention items: (nr. 1) restless (nr. 3) squirmy, fidgety, and (nr. 16) not able to concentrate

### Analyses

Equivalent statistical analyses were conducted separately for each cohort using SAS version 8.2 (SAS, Cary, NC, USA). The sum of the three core ADHD symptoms (restlessness, fidgetiness, and inattention) ranged from 0 to 6. We reported the mean sum score by gender across cohorts and examined possible differences using 95% confidence intervals (CI).

The cohorts differed on living conditions, maternal education (equivalent in Danish and Finnish cohorts, but higher in the Swedish) and on family structure at follow-up (Swedish and Danish were equivalent, but percentage of intact families was higher in the Finnish cohort). Such indices of disadvantage have been previously shown to relate to ADHD diagnosis [23]. Therefore, we stratified by these two potentially confounding variables. We checked whether prevalence of severe ratings differed across cohorts and genders after stratification. Maternal education was dichotomized into no university education vs. at least some university education (coded 0 or 1, respectively). Family structure was defined as either intact, i.e. continuously living within the original biological family unit (coded 0), or within a disrupted family (coded 1), i.e. either single-parent or reconstructed family unit including a step-parent.

Multivariate logistic regression models were used to assess the association between core ADHD symptoms and scholastic impairment adjusted for maternal education, family structure, and child gender. We examined whether the sum of symptoms, based on a continuous scale, was associated with impairment among boys and girls in each of the cohorts. We also report the strength of the associations using standardized estimates (called β). We then focused on the most severe ratings, i.e. *certainly true *because of their clinical relevance in relation to impairment. Core symptoms scores were dichotomized as severe ratings (coded 1) versus lower ratings (*somewhat true *or *not true*, coded 0). We combined the two hyperactivity symptoms and required a severe score on both restlessness and fidgetiness to code 1 and 0 represented any other combination. All classifications were done prior to analyses. Separate models were run for each type of symptom (predictor) and each scholastic skill (outcome).

## Results

The samples were based on the number of traceable live births in each cohort (rather than on the number of recruited pregnant women) for which parental consent to contact the teacher was obtained and consisted of a total of 13,087 children. Table 1 shows retention at follow-up was considerably lower in Sweden and Denmark (74% and 61%, respectively) than in Finland (92%). Maternal consent to contact teachers in Sweden was obtained for 79% and of these 96% of teachers participated. Attrition analyses for Sweden showed participants were similar to national averages on socioeconomic status and birth outcomes and permission to contact the teacher was not related to maternal ratings of child behavior, gender, and socioeconomic status [24]. In Denmark, 65% of parents provided permission to contact teachers and of these 85% participated. In Finland, the teacher questionnaires were originally sent to the parents who forwarded them to the teachers; 92% of the teachers responded. There were no differences in response rates according to neonatal risk [25].

The unadjusted mean sum score (95% CI) for the three hyperactivity-inattention symptoms for boys in Swedish, Danish, and Finnish cohorts were 1.9 (1.50, 2.30), 1.6 (1.47, 1.63), 1.3 (1.25, 1.35), respectively. The Finnish mean was significantly different from Swedish and Danish means at a probability level of at least .05. Girls' means were 0.7 (0.47, 0.93), 0.6 (0.51, 0.60), 0.5 (0.47, 0.53), respectively and did not differ significantly.

Unadjusted prevalence of high scorers on each of the inattention and hyperactivity core symptoms is presented in table 2 separately for cohort country and gender. A larger portion of boys received a severe score, i.e. a rating of "2" (*certainly true*) in comparison to girls. The percentage of boys receiving a high score on any core symptom was lowest in the Finnish cohort. Inattention was most prevalent for children in Denmark.

**Table 2:** Unadjusted percentages of children scoring high (rated as certainly true) on core symptoms per cohort country and gender

	**Boys**			**Girls**		
	Inattention	Fidgetiness	Restlessness	Inattention	Fidgetiness	Restlessness

Sweden	18.3	18.3	12.5	1.0	4.2	1.9
Age 7–8, 2001–2						
Denmark	21.5	9.3	13.1	7.4	1.6	2.8
Age 10–12 2001						
Finland	6.2	7.8	10.7	1.5	1.7	2.6
Age 7–8 1993–4						

Prevalence of hyperactivity symptoms did not differ significantly across cohorts after stratification by maternal education and family structure (detailed data available on request). Approximately 15–20% of boys from disadvantaged homes (low maternal education and disrupted families) were rated high on hyperactivity symptoms in all cohorts. Well below 10% of girls received such a rating. Less than 10% of boys and much less than 5% of girls in the most advantaged strata (high maternal education and intact families) were high on hyperactivity symptoms in all cohorts. However, inattention differed between cohorts. Fewer disadvantaged boys in the Finnish cohort received severe ratings on inattention than in the Danish, 10% vs. 34% respectively. Danish and Swedish cohorts did not differ. Inattention among girls was clearly most prevalent in the Danish cohort in comparison to Finnish cohort, 13% vs. 3%, and there were no differences between the Swedish and Finnish cohorts.

**Figure 1:**
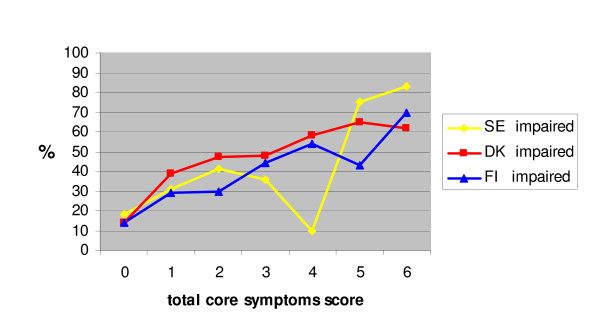
The portion of children with at least one scholastic skill impairment as a function of total core symptom score, SE impaired (yellow line), DK impaired (red line), FI impaired (blue line).

Twenty eight percent of children in the Swedish and Danish cohorts and 22% in the Finnish had impaired scholastic performance on at least one skill. Figure 1 shows the relation between total sum score on the core symptoms (range = 0–6) and the percent of children having at least one scholastic impairment. There was a positive relation in all cohorts. There was greater variability in the Swedish cohort in comparison to the others, presumably due to limited sample size.

In order to determine whether the association between the sum of the core symptoms and impairment applied only to particular skills, we examined the association with each skill separately in each cohort and for each sex. Further, we were also interested in whether the associations held for both boys and girls. Table 3 shows the results adjusted for maternal education, and family structure. Symptoms and impairment were related in all cohorts among both genders. Generally, for each unit increase in symptom scores there was approximately a 50% increase in the risk of impairment. Although, the associations were not significant for the most part in Sweden, as seen from the confidence intervals, the β values indicate that the associations were in the same direction as in the other cohorts. The association for girls seemed particularly weak in Sweden, but this could be explained by the low sample size that makes the estimates less accurate. The association was significantly stronger for girls in both Denmark and Finland. For example, Danish girls had a two-fold increase in risk of impairment in reading (95% CI: 1.8 – 2.2), whereas Danish boys had a 60% increase (95% CI: 1.5 – 1.7).

**Table 3:** Multiple logistic regression analyses for scholastic impairment by the sum of core ADHD symptoms^1 ^adjusted for maternal education^2 ^and family structure^3 ^for each cohort country

	**Boys**									**Girls**								
	Sweden			Denmark			Finland			Sweden			Denmark			Finland		

	β	OR	95% CI	β	OR	95% CI	β	OR	95% CI	β	OR	95% CI	β	OR	95% CI	β	OR	95% CI

Reading	.24	1.2	1.0, 1.6	.49	1.6	1.5, 1.7	.35	1.5	1.4, 1.5	.18	1.3	.8, 2.2	.41	2.0	1.8, 2.2	.33	1.7	1.6, 1.9
Writing	.48	1.5	1.2, 1.9	.36	1.4	1.3, 1.5	.34	1.4	1.4, 1.5	.15	1.3	.8, 1.9	.35	1.8	1.6, 1.9	.32	1.7	1.6, 1.8
Mathematics	.28	1.1	.9, 1.7	.44	1.5	1.4, 1.6	.40	1.5	1.4, 1.6	.15	1.3	.9, 1.8	.37	1.8	1.7, 2.0	.32	1.7	1.6, 1.8

^1^the sum of the symptoms ranged from 0–6

^2 ^No university education versus at least some university education

^3 ^Intact biological family versus disrupted (single-parent or step-parent present)

To assess whether severity in either type of symptom was related to impairment, we examined severe ratings as these may have more clinical relevance. Table 4 presents the association between severe ratings of inattention and hyperactivity (high score on both restlessness and fidgetiness) symptoms and each scholastic skill separately adjusted for gender, maternal education, and family structure. Both inattention and hyperactivity were strongly related to scholastic impairment. The relative strength of the associations was consistently stronger between inattention and impairment across all basic skills and cohorts in comparison with hyperactivity. The pattern of results was replicated in each of the cohorts for all of the skills.

**Table 4:** Multiple logistic regression analyses for scholastic impairment by inattention and hyperactivity^1 ^core symptoms adjusted for maternal education^2^, family structure^3^, and gender for each cohort country

	**Sweden**				**Denmark**				**Finland**			
	Wald χ^2^	p <	OR	95% CI	Wald χ^2^	p <	OR	95% CI	Wald χ^2^	p <	OR	95% CI

Reading impairment												
Inattention	6	.01	4.2	1.3, 13.0	469	.0001	10.5	8.5, 12.9	245	.0001	7.9	5.8, 9.5
Hyperactivity	1	.25	1.9	0.6, 5.7	118	.0001	3.8	2.9, 4.8	144	.0001	4.4	3.5, 5.7
Writing impairment												
Inattention	20	.0001	12.5	4.1, 38.1	309	.0001	5.4	4.5, 6.5	220	.0001	6.7	5.2, 8.7
Hyperactivity	11	.001	4.9	2.0, 12.5	73	.0001	2.6	2.1, 3.2	107	.0001	3.5	2.8, 4.5
Mathematics impairment												
Inattention	4	.05	4.0	1.1, 14.7	304	.0001	7.3	5.9, 9.2	318	.0001	10.6	8.2, 13.7
Hyperactivity	.5	.50	1.5	0.5, 5.1	72	.0001	3.1	2.4, 4.0	176	.0001	5.7	4.4, 7.4

^1 ^High score on both restlessness and fidgetiness (see Analyses section)

^2 ^No university education versus at least some university education

^3 ^Intact biological family versus disrupted (single-parent or step-parent present)

## Discussion

In general population based cohorts from three European countries including over 13,000 children, we found a strong and consistent pattern of associations between core ADHD symptoms and scholastic impairment. These results are in line with previous research showing comorbidity between ADHD and learning problems [26,27], and extend previous findings by confirming an association between core ADHD symptoms and impairment in general population samples. The same associations are similar to what has been shown for clinical cases of ADHD indicating that ADHD diagnoses are just extreme values from a continuous distribution (e.g. like hypertension).

Prior work has focused on literacy skills and found an association with ADHD [28]. However, we found that mathematics was also strongly associated with core symptoms, even when taking into account indices related to disadvantage (maternal education and family structure) and child gender. The association between inattention and scholastic impairment was more pronounced than the association between hyperactivity symptoms and impairment. Similarly, others [29] have found reading difficulties were more strongly associated with the ADHD inattentive than the hyperactive subtype.

Girls had a lower prevalence of severe symptoms in all cohorts, which is in line with the gender disparity in ADHD diagnosis. The larger cohorts showed significantly stronger relative associations between core symptoms and impairment among girls as compared to boys.

A major change in the DSM-IV from earlier versions was the addition of impairment requirement. Symptoms must generate impairment in order to be considered a psychiatric disorder. Several reports show that many children are impaired but do not reach the threshold number of symptoms and, therefore, do not meet the diagnostic criteria [30]. Impairment should weigh heavily in the diagnostic process even when children have fewer symptoms than required [31]. Our data do not allow us to identify children meeting the ADHD diagnosis and many of the children that scored high on the core symptoms we recorded most likely do not meet the full criteria. Still, children who scored high on the core symptoms and have impairment are in need of support whether or not they fulfill diagnostic requirements. Children with psychiatric symptoms are more likely to dropout of secondary school [32] and children with ADHD have poor long-term academic underachievement [33]. Further, scholastic impairment is related to delinquency [34,35]. Leaving children's needs unmet (behaviorally or pharmacologically) could result in many of them not reaching their full potential.

Initial screening procedures could be conducted in schools as teachers are in a good position to first detect learning problems and notice behavioral deviations because children with ADHD symptoms often disrupt the classroom. Ideally screening for ADHD symptoms could be a part of the school health surveys. Alternatively, teacher ratings on core symptoms could be incorporated into routine progress reports given to parents. Early support in the form of academic assistance, teaching coping strategies to children (e.g. organizational skills training), and teachers' monitoring behaviors in the classroom would be important first steps. This would also facilitate an alliance between teachers and parents to work on shared goals. This strategy is in keeping with a greater emphasis being placed on support rather than assessment [36]. Multimodal interventions alleviate symptoms and help in reducing impairment [37,38]. Until then, however, parents or clinicians should request this information from teachers and make it available when determining whether further evaluation is warranted. This study is in line with previous research showing the value of teacher and parent rating scales and indicates that teacher ratings of a few core symptoms and impairment are informative and can be an initial step in following the ADHD evaluation guidelines [39-41].

Prevalence of core symptoms was related to living conditions in all three cohorts. Our results converge with previous reports showing that disadvantaged children are more likely to receive high ratings on behavioral problems [42,43]. Thus, policy makers can increase surveillance of ADHD core symptoms and scholastic impairment among disadvantaged children who may be more vulnerable for negative outcomes.

The finding that inattention was lowest in Finland (even after stratification on living conditions) merits further research to discern whether etiological factors or other unmeasured factors related to living conditions are at the root of the observed difference. It is unclear to what extent methodological differences between cohorts could have contributed to the differences.

There are some limitations to consider. First, methodology differed somewhat between the cohorts e.g., data collection took place 6 years earlier in the Finnish cohort. This difference in time of data collection may explain why Finnish children were rated less inattentive than in the other two cohorts. It may be possible that awareness of ADHD symptoms has been steadily increasing within the last decade and may have affected teachers' willingness to endorse symptoms. However, it seems unlikely that increased awareness would pertain only to inattention and not to hyperactivity symptoms. A Swedish community study [44], which collected data at the time of the Finnish data collection (i.e. 6 years earlier), found similar prevalence rates as those presently reported for the Swedish cohort. Our Finnish prevalence rates are practically identical to results found from a later date from a study conducted in another part of Finland [45]. Because Swedish and Finnish children were the same age, differences in inattention rate cannot be attributed to developmental effects. Thus, neither developmental differences nor time effects between cohorts can fully explain prevalence differences.

Second, we were limited to teacher-reported core symptoms and scholastic impairment. It may be that teachers tend to rate a child poorly in one area if he or she is rated poorly in another or that behavioral ratings bias perceptions of scholastic performance, e.g. due to a negative halo effect. However, teachers within each cohort rated various behaviors and outcomes pertaining to well-being and development, consequently the connection between the three core symptoms that we study and scholastic impairment was not likely to have been made. The majority of children in our study were first-graders, therefore, official grades or national test scores are not available with which to compare teacher ratings. Nonetheless, our measure has ecological validity as teachers are in the best position to rate academic performance and do so in reality. Teacher ratings of behavior have been found to reflect age-appropriate evaluations [46]. Symptoms may present differently according to environment and it is suggested that inattention and hyperactivity symptoms are more reliably observed in a school setting [41]. In this respect, core ADHD symptoms at school may be more relevant for scholastic impairment than in other environments. Recently, Caroll and colleagues [28] found that child literacy impairments were related equally to both teacher and parent ratings of ADHD symptoms using the SDQ. Further, there is a school effect related to child performance [47], however, teachers in our samples were not confined to any particular school, but came from large geographic areas.

Third, we related only three core symptoms to scholastic performance rather than clinical diagnosis. Our results show that functional impairment is not only limited to children who fulfill all the clinical criteria for ADHD, but suggest that increases in symptomatology and impairment go hand-in-hand in the general population.

Fourth, we used cross-sectional data although we expect a relation between ADHD symptoms and scholastic impairment to develop over time as has been suggested in clinical studies [3,48]. It is likely that there is a dual pathway connecting behavioral symptoms and scholastic difficulties. Our follow-ups were initiated during the acquisition of scholastic skills (Swedish and Finnish cohorts). Thus, there may be a greater likelihood for the causal pathway to be from hyperactivity-inattention symptoms to scholastic deficits, which is consistent with findings showing inattention symptoms contribute to later reading difficulties [49].

## Conclusion

Despite these limitations, the relation between hyperactivity and inattention symptoms and scholastic impairment was replicated in three general population samples of nonreferred children from different countries, which indicates a robust relation. Symptoms were related to all the skills we investigated suggesting overall scholastic difficulties. Scholastic impairment is a powerful predictor of adverse developmental trajectory.

## Competing interests

The author(s) declare that they have no competing interests.

## Authors' contributions

AR participated in the design of the study and drafted the manuscript. KP, JM, and AR performed the statistical analyses. CO provided the Danish, AT and IM the Finnish, and AR the Swedish cohort datasets. JO and TBH, MRJ, and AR supervised the cohort studies in Denmark, Finland, and Sweden respectively. All authors contributed to the manuscript, read and approved the final version.

## Pre-publication history

The pre-publication history for this paper can be accessed here:


